# The Double-Fortified Salt (Iodized Salt with Iron) Consultation: A Process for Developing Evidence-Based Considerations for Countries

**DOI:** 10.1093/jn/nxaa157

**Published:** 2021-02-15

**Authors:** Robin Houston, Becky L Tsang, Jonathan Gorstein

**Affiliations:** Iodine Global Network, Seattle, WA, USA; Iodine Global Network, Seattle, WA, USA; Iodine Global Network, Seattle, WA, USA

**Keywords:** fortification, salt, iodization, iodine, iron

## Abstract

The addition of iodine to edible salt has been one of the most important public health successes of the past half century, enabling most countries to achieve optimal iodine intake and protect the brains of unborn children from the adverse consequences of iodine deficiency. Salt has been an ideal vehicle for this effort because of its near universal and narrow range of consumption, relative ease of technology for salt iodization, and capacity for virtually all salt producers to add iodine. As a result of the success of salt iodization, there has been growing interest in using salt as a vehicle for other important micronutrients, particularly the addition of iron to iodized salt to produce double-fortified salt (DFS), to combat the persistent problem of iron deficiency and iron deficiency anemia. Because of this growing interest in DFS and the need for a comprehensive review of evidence to support the viability of this intervention, the Iodine Global Network (IGN) initiated a global consultation to gather all available data on different aspects of DFS. IGN identified 4 key areas considered essential to understand for a successful fortification intervention: *1*) efficacy and effectiveness, or how well DFS produces a health impact in controlled and real-life settings; *2*) technical considerations for production, or what are the minimum requirements to manufacture DFS; *3*) program implementation to describe experiences thus far with the delivery of DFS across multiple platforms; and *4*) comparison of DFS with other iron fortification efforts to determine the comparative advantage of DFS to improve iron intake and prevent iron deficiency anemia. This preface provides an overview of the DFS Consultation objectives, process, and objectives.

The addition of a small amount of iodine to edible salt has been one of the most important public health successes of the past half century, enabling most countries to achieve optimal iodine intake and protect the brains of unborn children from the adverse consequences of iodine deficiency (1). Salt has been an ideal vehicle for this effort because of its near universal and narrow range of consumption, relative ease of technology for salt iodization, and capacity for virtually all salt producers to add iodine. As a result of the success of salt iodization, there has been growing interest in using salt as a vehicle for other important micronutrients, particularly the addition of iron to iodized salt to produce double-fortified salt (DFS), to combat the persistent problem of iron deficiency and iron deficiency anemia.

Because of this growing interest in DFS and the need for a comprehensive review of evidence to support the viability of this intervention, the Iodine Global Network (IGN) initiated a global consultation to gather all available data on different aspects of DFS. IGN identified 4 key areas considered essential to understand for a successful fortification intervention: *1*) efficacy and effectiveness, or how well DFS produces a health impact in controlled and real-life settings; *2*) technical considerations for production, or what are the minimum requirements to manufacture DFS; *3*) program implementation to describe experiences thus far with the delivery of DFS across multiple platforms; and *4*) comparison of DFS with other iron fortification efforts to determine the comparative advantage of DFS to improve iron intake and prevent iron deficiency anemia.

IGN reached out to a number of key partners and experts in salt and iron fortification to identify and form a steering group to define the scope of the consultation, provide an objective review of the evidence across those 4 key areas collected, and develop a consensus summary of the evidence and critical considerations for countries interested in DFS. With steering group guidance, IGN facilitated the consultation by commissioning authors for each of these topics and worked with them to develop a series of background papers, which are included in this supplement. IGN and the steering group reviewed and provided feedback at several stages of each paper's development.

This supplement includes an introduction that provides the technical background on iron fortification and the issues that affect the likelihood of health impact and operational considerations when iron is added to a food vehicle (2). The objective of DFS is to improve nutrition status, specifically of iron and iodine. To review the efficacy and effectiveness of DFS on biomarkers of iodine and iron status, Larson et al. conducted a systematic review and meta-analysis (3), comparing the main DFS formulations that have been researched. Outcomes assessed include anemia, hemoglobin, ferritin, iron deficiency anemia, urinary iodine, cognitive measurements, child development, and any reported adverse outcomes. This review updates and expands upon a recently published systematic review and meta-analysis (4).

Primary research, in the form of key informant interviews and visits to salt production facilities in India (where the majority of DFS is produced globally), was used to gather evidence on the technical requirements and private sector experiences in the production of DFS (5). Shields and Ansari (5) explore the minimum requirements a producer must meet in order to manufacture a high-quality DFS product that minimizes any potential sensory changes and iodine losses, including the input salt standards in production, iron formulation quality, blending, and packaging considerations. This paper also explores the initial capital investment and ongoing costs for DFS compared with salt iodization.

A similar approach was used to gather details on pilot programs and ongoing efforts to introduce DFS globally (6). With only 2 salt manufacturers currently providing DFS in the retail market, this paper focuses primarily on experiences in social safety net programs in India, where the largest distribution of DFS occurs through government programs aimed at improving nutrition in vulnerable populations.

Finally, a desk review and literature summary was undertaken to compare DFS with other iron fortification vehicles (7). The review summarizes the experience with iron fortification of other foods, including wheat flour, maize flour, rice, and milk. The paper compares bioavailability of iron compounds across the food vehicles and discusses the most suitable iron compounds for each food; issues with sensory changes after fortification and how these are addressed; consumption patterns; and the landscape for industrial food processing for these foods, which directly affects ease of implementation.

All of the papers touch on the issue of organoleptic changes. With the currently available iron formulations, adding iron to iodized salt has been shown to consistently cause color changes to the DFS product and risks iodine loss due to interactions with iron. There is a higher risk of color changes and iodine losses especially when the iodized salt used in DFS is low purity and has high moisture, or when salt is stored in moist conditions. These changes can potentially reduce iodine intake if they affect acceptance of DFS or cause the consumer to choose a nonfortified salt, thus posing a potential threat to the success achieved thus far in universal salt iodization. These organoleptic changes are discussed in each paper, reviewing whether they affect health impact, technical production, or program implementation.

To discuss the evidence available for DFS, IGN initiated 2 consultation meetings. The first, held in April 2019, included authors and steering group members and was designed to provide a forum for discussion of the evidence emerging for each of the 4 topics and provide feedback to authors. The second, involving only steering group members, took place in December 2019 and was designed to review the evidence, define areas of agreement on the evidence, and draft a consensus brief that would summarize the evidence and provide policy makers key considerations for DFS. This brief, coauthored by the steering group, is meant to provide policy makers who are considering DFS with information to guide their decision making. The brief was not designed to provide specific recommendations but, rather, to help determine what information policy makers should be aware of in order to assess whether DFS is a viable intervention in their country.

It is the hope of the steering group that this supplement will provide a comprehensive evidence base that can help policy makers in countries considering initiating DFS so that they may make informed decisions for their national nutrition strategy.

## Notes

Published in a supplement to *The Journal of Nutrition*. The Guest Editor for this supplement was Becky L Tsang. Publication costs for this supplement were defrayed in part by the payment of page charges by the Iodine Global Network. The opinions expressed in this publication are those of the authors and are not attributable to the sponsors or the publisher, Editor, or Editorial Board of *The Journal of Nutrition*.

The Supplement Coordinator for the supplement publication was Becky L Tsang.

This consultation was made possible through funding support from UNICEF (through a grant from USAID) and the Bill & Melinda Gates Foundation grant OPP1195090.

Author disclosures: The authors report no conflicts of interest.
